# Investigating surgical smoke in otolaryngology operating rooms

**DOI:** 10.1038/s41598-022-05701-1

**Published:** 2022-02-02

**Authors:** Chun-I. Li, Ying-Hsiang Chou, Jar-Yuan Pai, Chih-Hsuan Chen, Min-Chi Chiang

**Affiliations:** 1 Department of Otolaryngology, Chang Bing Show Chwan Memorial Hospital, Changhua County, 50544 Taiwan, ROC; 2 Department of Radiation Oncology, Chung Shan Medical University & Chung Shan Medical University Hospital, Taichung City, 40201 Taiwan, ROC; 3 Department of Health Policy and Management, Chung Shan Medical University & Chung Shan Medical University Hospital, Taichung City, 40201 Taiwan, ROC; 4 Department of Health Policy and Management, Chung Shan Medical University, Taichung City, 40201 Taiwan, ROC

**Keywords:** Health care, Health occupations

## Abstract

Surgical smoke is a common chemical hazard produced from the use of electrocautery, laser, or ultrasonic scalpels during surgery. It has been proved harmful to medical personnel. Thus, it is important to monitor surgical smoke concentrations in the operating room. In the past decade, many researches regarding surgical smoke were discussed in different professional healthcare fields, but few showed the correlation between surgical smoke and otolaryngology surgery. In this study, the concentrations of particulate matter and formaldehyde were measured during thirty cases of several types of otolaryngology surgery in a regional research hospital in Taiwan. The concentrations of 0.3 µm and 0.5 µm particulate matter raised rapidly in the main knife range at the beginning of the electrocautery knife used, and then decreased by half after 5–10 min of use. The concentrations of formaldehyde were ranged from 1 to 2 ppm during the surgery, which is higher than the permissible exposure limit. While many medical staffs are working in the operating room and are exposed to the smoke hazard, effective strategies for collecting and eliminating the smoke should be taken in all medical facilities.

## Introduction

Public health issues related to health promotion have always been taken seriously. In addition, the awareness of occupational safety is on the rise. Usually, cross-infection between patients are discussed frequently in medical institutions. However, the risks of exposure that medical personnels suffered from in hospitals are seldom discussed, according to studies by Bell and Wendt^1,2^. The U.S. Centers for Disease Control and Prevention (CDC) categorized the major exposure faced by the healthcare workers into Infectious Agents, Chemical Hazards, and Physical Hazards^3^. The exposure to infection risk of healthcare workers have been emphasized in many studies, especially during the COVID-19 pandemic^4^. Discussion of robotic technology used in surgical environments was taken seriously to minimize contact between patients and healthcare providers^5^. By contrast, the chemical hazards may not draw as much attention as infection risk.

Surgical smoke is one of the most common chemical hazards produced from the use of electrocautery, laser, or ultrasonic scalpels in the operating room (OR). Each year, about 500,000 surgeons, nurses, anesthesiologists, and technicians in American are exposed to the smoke, as reported by the Occupational Safety and Health Administration (OSHA)^6^. However, there are still many medical staff lacking awareness of prevention regarding surgical smoke. Michaelis et al. found that only 51% of surgeons in hospitals and 70% of the surgeons in outpatient facilities “mostly” or “always” take notice of protecting themselves from the hazard of surgical smoke in their survey^7^. Surgical smoke is generated by the use of diathermy devices during open surgery as well as endoscopic surgery. It is composed of about 95% steam and 5% particulate matter (PM) from the decomposition of tissue and blood at high temperatures. The composition varied with different types of surgeries, the instrument used, and the tissue that was cauterized^8,9^. The potentially carcinogenic components within surgical smoke are sufficiently small and respirable^10^. According to Bigony and Ko, the gaseous substances in the smoke, including toluene, benzene, formaldehyde, and polycyclic aromatic hydrocarbons (PAHs), are harmful to the human body, and most of them are close to or exceed the limitation set by the OSHA^11,12^.

It has been proved that surgical smoke is harmful to medical personnel working in the OR^13^. Cheng et al. concluded that the particle sizes of surgical smoke are less than 1 μm mostly, and the average aerodynamic diameter of PM generated by electrosurgical tools is about 0.07 μm^14^. These hazardous substances can be inhaled and deposit on the upper respiratory tract, then cause respiratory diseases like chronic obstructive pulmonary disease, asthma, bronchitis, and even lung cancer^15^. Formaldehyde is one of the common compounds found in surgical smoke. Formaldehyde not only can irritates eyes, nose, and respiratory system, but also cause cough and bronchospasms^16^. It has been classified as a Carcinogenic to humans (Group 1) by the International Agency for Research on Cancer (IARC)^17^. The awareness of taking precautionary measurements is on the rise. Simple and effective methods were developed to exhaust smoke during surgical operations, such as using a synchronous smoke evacuator near the electric scalpel^18^.

In the past decade, many researches regarding surgical smoke were discussed in different professional healthcare fields, such as surgical smoke exposure under plastic surgeries^19^ and dermatologic procedures^20^, human papillomavirus (HPV) transmission and HPV-related disease following surgical smoke exposure^21^, or how coronavirus with regards to its hazards within surgical smoke and the procedures that could mitigate the potential risks to healthcare staff^22^. Among these studies, only few showed the correlation between surgical smoke and otolaryngology. The latest related study was conducted by O’Brien et al., assessing the exposure of surgical personnel to known carcinogens during pediatric tonsillectomy and adenoidectomy^23^. In order to conduct further study in other common otolaryngology procedures, this study aimed to investigate the surgical smoke that medical staff are exposed to during several types of otorhinolaryngology surgery, including submandibular gland excision, neck mass excision, tonsillectomy, and functional endoscopic sinus surgery. In this study, suspended particulates of 0.3 μm, 0.5 μm, and 5 μm, as well as formaldehyde were detected at different location and time points. The surgical smoke was detected inside and outside the operating rooms to evaluate the high concentration area. The different detection time was to measure the variation of the concentration during the surgery. Finally, the methods of smoke collection and removal are discussed to identify the appropriate strategies to protect the occupational safety of medical personnel.

## Methods

**Figure 1:**
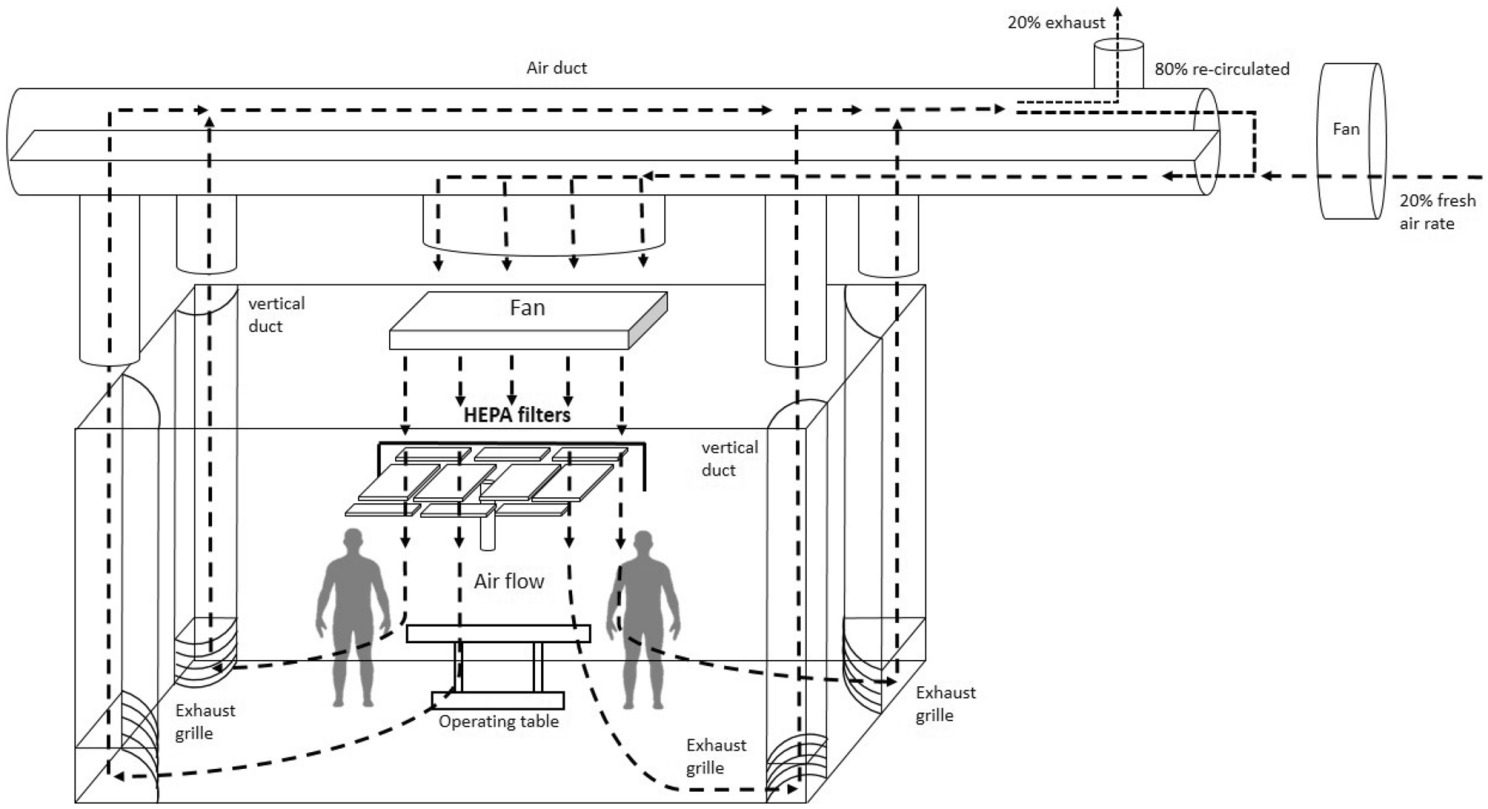
Schematic of air flow direction, air inlet and exhaust locations inside the operating room.

**Figure 2:**
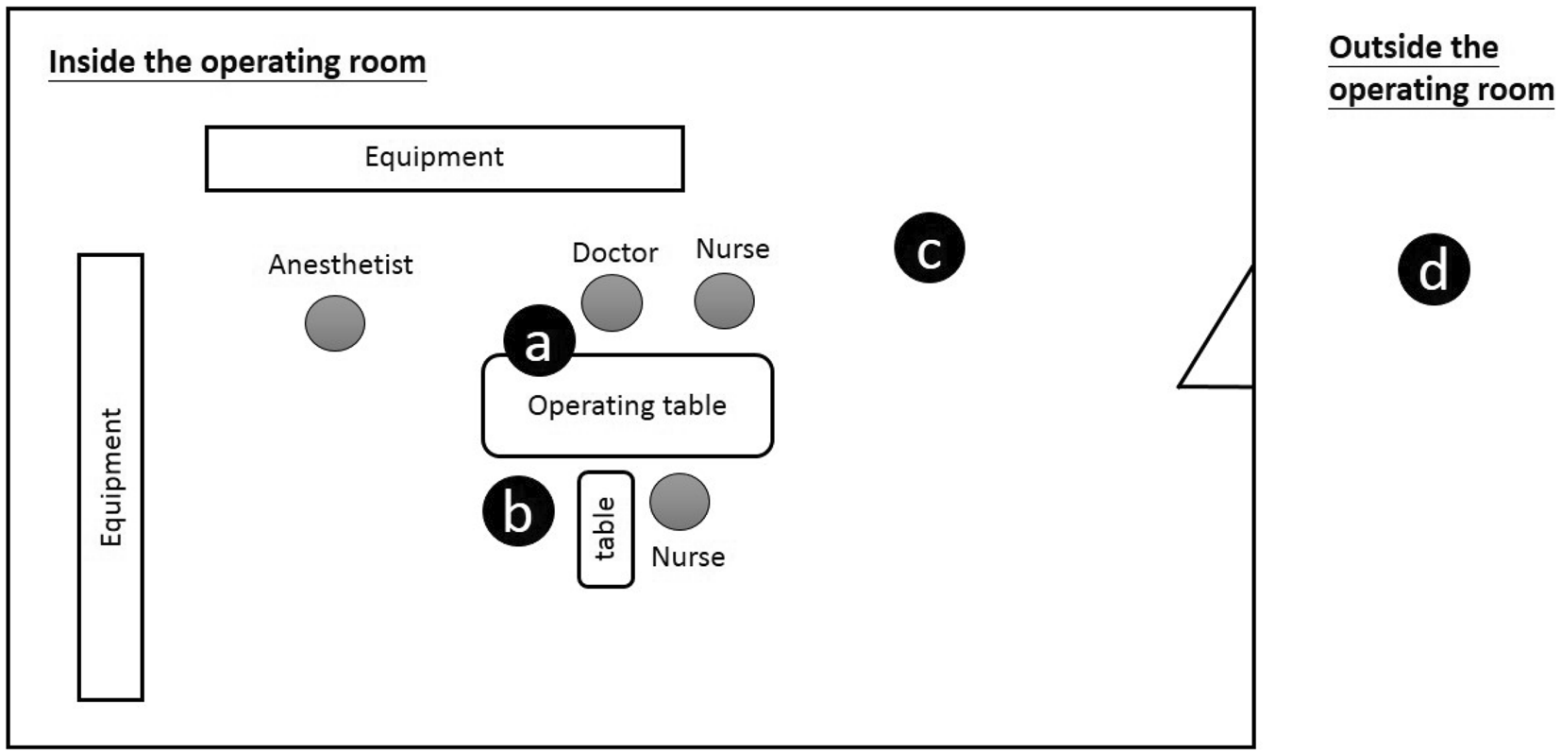
Schematic of the sampling locations in the OR. (**a**) The main knife range; (**b**) outside the operating table; (**c**) background; (**d**) outside the operating room.

This study was conducted at Chang Bing Show Chwan Memorial Hospital, a regional research hospital with 1400 beds in Taiwan. A total of 30 cases performed otorhinolaryngology surgeries by the same doctor and surgical team were selected. There were 1 doctor, 2 nurses, 1 anesthetist, and 1 staff member who was responsible for detecting the concentration of particles in each surgery. The surgical types included submandibular gland excision, neck mass excision, tonsillectomy, and functional endoscopic sinus surgery, which were all endoscopic surgeries. A monopolar or bipolar electrosurgical unit was used in each surgery. The surgery time ranged from 1 to 3 h, the average operation time was 1.5 h. The ambient temperature in the OR was maintained between 20 and 22 °C and ambient humidity is kept steady around 30–60%. Heat load is 10–25 RT (1 RT = 3.516 kW). The operating room with the same ventilation conditions were used. The ventilation rate was kept at 20 to 25 air changes per hour. The location of air inlet was set directly above the operating table. The air flow went through HEPA (high efficiency particulate air) filters, 10 flat pieces in total and the flat dimension is 60 cm × 120 cm each. The exhaust locations were the four vertical ducts in all corners of the operating room. Figure 1 showed the schematic of air flow direction, air inlet and exhaust locations inside the operating room. The fresh air rate introduced into OR is 20% and the re-circulated air ratio is 80%. The concentrations of particulate matter and formaldehyde were measured from different sampling locations and sampling time were set for 1 min each time. Figure 2 showed the schematic of sampling locations, which were to measure the concentrations of (a) the main knife range, (b) outside the operating table, (c) background, and (d) outside the operating room. The distance between location (a) and the operating table is 15–20 cm. This distance is equivalent to the distance between the medical staff and the operating table during the operation. Statistical analysis of the measurement data was conducted with the Statistical Analysis System (SAS) for Windows V9.4.

### Particulate matter

The suspended particulate matter was detected using a direct-reading instrument, a Handheld Laser Particle Counter (HAL-HPC300) made by Hal Technology, Fontana, U.S.A. Three months before this study started, the instrument was sent back to the original equipment manufacturer (OEM) for calibration. Based on digital signal processing, the basic principle of HAL-HPC300 is that the laser scattering pulse signal of an aerosol particle output from an optical sensor is processed and counted. It can simultaneously measure three channel sizes of particulate matter that are configurable by users^24^, which was set as 0.3 μm, 0.5 μm, and 5 μm in this study. The setting of sizes measurement complied with the Federal Standard 209E^25^. The measurement range of HAL-HPC300 is 0.3–10 μm and the coincidence loss is less than 5% at 2,000,000 particles/ft^3^. The detection unit of the particulate matter was CU.FT (particles/cubic feet). The detection times were ‘before the operation’, ‘the beginning of the electrocautery knives used’, ‘5 to 10 min after the electrocautery knives used’, and ‘the end of the operation’. The sampling period was one minute each time. In the beginning of the operation, the measurement of the main knife range and outside the operating table were conducted simultaneously to detect the concentration of particulate matter.

### Formaldehyde

Formaldehyde was detected using a real-time formaldehyde detector (model: HFX205) made by HAL Technology, Fontana, U.S.A. The detection range of the detector is 0 to 10 parts per million (ppm), the precision is 0.02 ppm, and the accuracy is ± 0.03 ppm (0–1.00 ppm). The sensor is electrochemical and the sampling method is a built-in pump. The air can be drawn in continuously at about 300c.c per minute for testing, and the detection unit is ppm. The detection locations and times were the same as those used in the method to detect particulate matter.

## Results

### The concentration of the particulate matter

**Figure 3:**
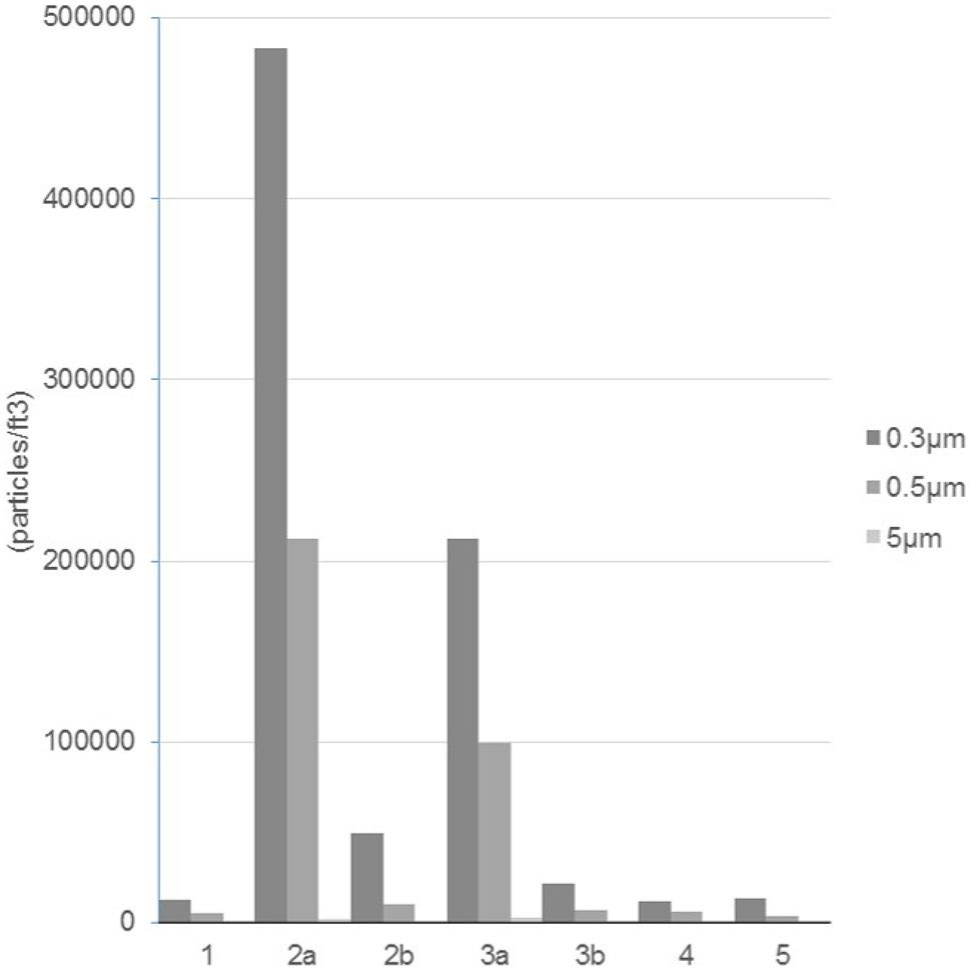
The variation in the average concentration of the PM. Horizontal axis: 1. Before the operation; 2a. Beginning of the operation in the main knife range; 2b. Beginning of the operation outside the operating table; 3a. After 5–10 min in the main knife range; 3b. After 5–10 min outside the operating table; 4. At the end of the operation; 5. Outside the operating room.

Figure 3 shows the variation in the concentration of particulate matter inside and outside the OR during the surgeries. In the OR, the average concentrations of particulate matter of size 0.3 μm and 0.5 μm reached their highest levels at the beginning of the electrocautery knife used at the main knife range. The median value and interquartile range of particulate matter measured is presented in Table 1. Compared with those detected at the beginning of the electrocautery knife used, the average concentrations of 0.3 μm and 0.5 μm particles become much lower and the concentration of 5 μm particles is a little higher after 5 to 10 min. Not surprisingly the concentrations of the particles at point a (the main knife range) were much higher than those detected at point b (outside the operating table).

**Table 1:** Median value and interquartile range of particulate matter measurements.

Sampling location and time	Particulate matter size	Median value	Interquartile range
Before the operation (1)	0.3 μm	8249	8653
	0.5 μm	4725.5	2738.5
	5.0 μm	551.5	580
Beginning of the operation in the main knife range (2a)	0.3 μm	11,857	181,622.2
	0.5 μm	2716	68,040.3
	5.0 μm	254	764.5
Beginning of the operation outside the operating table (2b)	0.3 μm	8602.5	19,953.8
	0.5 μm	3551.5	6085
	5.0 μm	212	587.3
After 5–10 min. in the main knife range (3a)	0.3 μm	9112	4544.3
	0.5 μm	2971	11,241.8
	5.0 μm	226	303.3
After 5–10 min. outside the operating table (3b)	0.3 μm	4853	12,677.8
	0.5 μm	2023	2660.5
	5.0 μm	254	255
At the end of the operation (4)	0.3 μm	7683	9289.8
	0.5 μm	3707	4726
	5.0 μm	368	737.8
Outside the operating room (5)	0.3 μm	4966.5	4839.3
	0.5 μm	2532.5	1903.8
	5.0 μm	339	227

Unit: particles/ft^3^.

A significant difference (p < 0.05) in 0.3 μm particulate matter between the beginning of the electrocautery knife used and 5–10 min after that at the main knife range was found. There were also a notable diversity (p < 0.1) in 0.3 μm particulate at different detection time outside the operation table. (Table 2) The difference in the concentrations of 0.3 μm and 0.5 μm particulate matter detected at the beginning of the operation and after the electrocautery knife used for 5–10 min was obvious, but this was not the case for 5.0 μm particulate matter (Table 3).

**Table 2:** The analysis of the concentration number of particulate matter detected during the use of electrocautery knife at different time points.

Sampling location	Particulate matter size	Detection time	Mean	Standard deviation	p-value
Main knife range	0.3 μm	Beginning of the knife used	4.83*10^5^	1.51*10^6^	*0.050
		5–10 min after beginning	2.12*10^5^	7.83*10^5^	
	0.5 μm	Beginning of the knife used	2.12*10^5^	8.21*10^5^	0.133
		5–10 min after beginning	9.96*10^4^	3.60*10^5^	
	5.0 μm	Beginning of the knife used	1.80*10^3^	4.58*10^3^	0.304
		5–10 min after beginning	2.97*10^3^	1.33*10^4^	
Outside the operation table	0.3 μm	Beginning of the knife used	4.97*10^4^	8.70*10^4^	^†^0.067
		5–10 min after beginning	2.13*10^4^	6.47*10^4^	
	0.5 μm	Beginning of the knife used	1.02*10^4^	1.38*10^4^	0.165
		5–10 min after beginning	6.48*10^3^	1.82*10^4^	
	5.0 μm	Beginning of the knife used	3.91*10^2^	4.67*10^2^	0.200
		5–10 min after beginning	5.30*10^2^	7.73*10^2^	

Unit: particles/ft^3^.

^†^0.05 < p < 0.1,*p < 0.05.

**Table 3:** The analysis of the concentration number of particulate matter detected during the use of electrocautery knife at different locations.

Detection time	Particulate matter size	Sampling location	Mean	Standard deviation	p-value
Beginning of the knife used	0.3 μm	Main knife range	4.83*10^5^	1.51*10^6^	^†^0.061
		Outside the operation table	4.97*10^4^	8.70*10^4^	
	0.5 μm	Main knife range	2.12*10^5^	8.21*10^5^	^†^0.096
		Outside the operation table	1.02*10^4^	1.38*10^4^	
	5.0 μm	Main knife range	1.80*10^3^	4.58*10^3^	^†^0.054
		Outside the operation table	3.91*10^2^	4.67*10^2^	
5–10 min after beginning	0.3 μm	Main knife range	2.12*10^5^	7.83*10^5^	^†^0.094
		Outside the operation table	2.13*10^4^	6.47*10^4^	
	0.5 μm	Main knife range	9.96*10^4^	3.60*10^5^	^†^0.083
		Outside the operation table	6.48*10^3^	1.82*10^4^	
	5.0 μm	Main knife range	2.97*10^3^	1.33*10^4^	0.163
		Outside the operation table	5.30*10^2^	7.73*10^2^	

Unit: particles/ft^3^.

^†^0.05 < p < 0.1,*p < 0.05.

### The concentrations of formaldehyde

**Figure 4:**
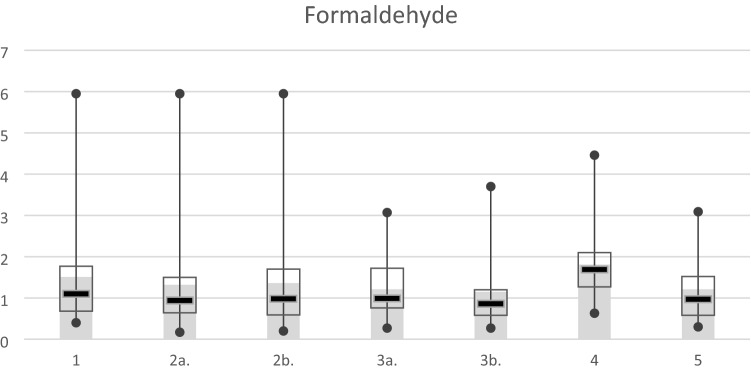
The variation in the average concentration, median value, and interquartile range of the formaldehyde. The horizontal axis is the same as that in Fig. 3.

Figure 4 shows the detection results of formaldehyde at different locations. The concentration detected in the operating room before the use of the electrocautery knife was 1.51 ppm. The concentrations detected at the beginning of the knife used in the main knife range and outside the operation table were a little lower than those detected before the knife used. After 5–10 min, the concentration decreased to 1.21 ppm and 1.15 ppm respectively. At the end of the operation, the concentration of formaldehyde was the highest among all. The corresponding concentration outside the operating room was the same as that detected at the knife used after 5–10 min in the OR.

A notable diversity (p < 0.1) between the beginning of the operation and 5–10 min after beginning at the periphery of the operation table was found. The p-values relating to this difference for formaldehyde was 0.068. The concentrations detected in the main knife range at different times were not significantly different. (Table 4) There was also no significant difference between the main knife range and outside of the operation table while the measurement was taken at the same time. (Table 5).

**Table 4:** The analysis of the concentration number of formaldehyde detected at different time points.

Sampling location	Detection time	Mean	Standard deviation	p-value
Main knife range	Beginning of the knife used	1.32	1.20	0.206
	5–10 min after beginning	1.21	0.69	
Outside the operation table	Beginning of the knife used	1.36	1.24	^†^0.068
	5–10 min after beginning	1.15	0.87	

^†^0.05 < p < 0.1,*p < 0.05.

**Table 5:** The analysis of the concentration number of VOC and formaldehyde detected at different locations.

Detection time	Sampling location	Mean	Standard deviation	p-value
At the beginning of the knife used	Main knife range	1.32	1.20	0.326
	Outside the operation table	1.36	1.24	
5–10 min after beginning	Main knife range	1.21	0.69	0.270
	Outside the operation table	1.15	0.87	

## Discussion

In this study, we found a rapid elevation in the concentrations of 0.3 μm and 0.5 μm particles at the beginning of the electrocautery used. Five to ten minutes after the beginning, the concentrations of 0.3 μm and 0.5 μm particles decreased by half, though only 0.3 μm particles showed a significant difference. It confirm to the results of Brüske-Hohlfeld et al., a very high concentration of particles was confined to the surrounding area of the operating side and was present for only a few minutes with an efficient air conditioning system^9^. We also noticed that 5.0 μm particles slightly increased after 5–10 min of the knife used both in the main knife range and outside the operating table, but the differences were not significant.

In this study, the highest concentration of 0.3 μm and 0.5 μm particles were measured at the beginning of the operation in the main knife range. However, in another correlated study, characterization of smoke generated during the use of surgical knife in laparotomy surgeries, the results of the highest peak was different. It stated that the highest concentration of 0.3 μm and 0.5 μm particles were measured at the time point of “after 5–10 min of using the electrocautery knife” in the main knife range^26^. We thought the reasons for the difference is that the laparotomy surgeries were performed in abdominal cavity or pelvic cavity which is a relative enclosed space compare to the operation area of otolaryngology surgery. The smoke may accumulated in the cavity at the first time and diffused from the hole with the endoscopy moved in or out in the process of surgery.

In Fig. 4, the formaldehyde concentration was already high (1.51 ppm) before the operation. It was considered to be the residues of the previous operation. According to the UN World Population Prospects 2019, Taiwan’s population density (673.7 people per sq. km) is the second greatest in the world^27^. Due to the high population density and medical treatment culture, the high utilization rate of OR is a common situation in Taiwan’s medical facilities, especially in crowded cities. In the hospital where this study was performed during prime time, 5–6 operations were conducted a day in a single OR. The tight schedule edged out preparation times between operations which also shorten the time of air circulation. Thus, the high formaldehyde concentration before the operation was considered to be the residues of the previous operation. This finding showed that the utilization rate of the OR is also a concerned factor when it comes to reducing surgical smoke. Even though extensive OR utilization is usually the goal of OR director or hospital administration, the environmental condition should also be considered when defining the efficiency of OR utilization.

One of the reasons that PM concentration decreased dramatically at the end of the operation (Figs. 3 and 4) but formaldehyde concentration increased (Fig. 4) on the contrary might be the effect of using HEPA filters in the ventilation system. HEPA filters are more effective for filtering PM than chemical materials such as formaldehyde. Other possible reasons causing formaldehyde concentration to rise at the end of the operation requires further study to find out.

Surgical smoke came from the momentary usage of laser scalpel. The generation of surgical smoke was non-continuously. Moreover, the variation of concentration changed rapidly due to different surgical procedures and scalpel materials used. Thus, the data dispersion were widely spread which led to high standard deviations. This result shows the fact that surgical smoke was not a steady value but variated momentarily. For instance, the raw data of 0.5 μm particle measurement detected ranged from 56 to 9027 (particles/ft^3^) in a single operation. Therefore, no error bars or standard deviation bars were shown in the figures since the bars cannot present the characteristic of surgical smoke in this study. However, the result also showed the limitation of this study. Limited by the measurement method and instrument used, this study failed to present continuous data throughout the whole surgery to show temporal variation. The limitation can be fixed in future related study.

The concentrations of formaldehyde detected in this study were ranged from 1 to 2 ppm, which is higher than the permissible exposure limit (PEL) an eight -hour time-weighted average (TWA), 0.75 ppm, given by the Occupational Safety and Health Administration (OSHA)^28^. Thus, it is very important to provide the protective equipment for medical personnel to prevent the hazards of surgical smoke. Another finding in this study is that the highest concentration of formaldehyde was detected at the end of the operation. More details of the conditions in the OR needed to be clarify to find out the reasons. A continuous measurement may be helpful to understand the variation of the concentration. The ventilation system may also played an important role to affect the concentration of surgical smoke.

Although some hospitals have installed smoke extraction systems or ventilation systems in the operating room, there are still many hospitals that are not aware of the use of suitable smoke extraction facilities. In the survey of Michaelis’s research, only 52% of hospital respondents and 65% of outpatient respondents reported using any suction system to capture surgical smoke^7^. Besides, some healthcare facilities purchased air purification systems in order to comply with regulatory requirements, but few of them bought consumable materials to maintain the system^26,29^. The current situation of medical sites presents the problem of insufficient environmental protection which must be strengthened and implemented. Possible strategies such as set up effective OR ventilation systems, wearing surgical mask or N95 mask, and using PTFE-coated blades are suggested in the following paragraphs.

Schultz suggested that central evacuation systems should be installed in hospital operating rooms, and a control panel should be equipped to adjust the flow rate and discharge the air in the operating room in a large range^30^. The smoke extraction system should have a High-Efficiency Particulate Air filter (HEPA filter), which has the best effect. The minimum effect of this filter is 99.97% for the removal of 0.3 μm particles and 99.99% for the removal of 0.5 μm particles, which can ensure that microparticles and microorganisms are removed effectively. An Ultra-Low-Penetration Air filter (ULPA filter) which can filter 0.1–0.2 μm particles with filtration efficiency up to 99.999% could also be considered^31^. In addition, a combination of HEPA or ULPA filters with activated carbon which can eliminate about 85% of volatile organic compounds (VOCs) in surgical smoke are available to use^32^.

A local exhaust ventilation device is a highly efficient smoke removal device that uses a hood close to the source of pollution to draw in the harmful smoke. The smoke is piped through an air cleaner device. The filtered air is exhausted by the fan and transported to the stack to discharge outdoors. Different ventilation system types also affect the efficiency of surgical smoke collection. In Romano’s research, the unidirectional downward airflow (UDV) system was more efficient in evacuating the smoke near the surgical area than upward displacement airflow (UWD) systems due to its large airflow volume and well-defined airflow pattern^33^.

The diameter of particulate matter in surgical smoke ranges from tens of nanometers (nm) to several microns (μm). It is suggested that healthcare workers in medical institutions should wear a respiratory protective equipment for their own health. The masks used in operations are made of three layers. The outer layer has the function of preventing dust and water from entering the mask. The middle and the inner layers have the function of absorbing moisture and droplets from the mouth and nose. Although the middle layer can effectively block more than 90% of 5 μm particulate matter, it cannot intercept 0.3 μm particulate matter^34^. A study by Alp et al. pointed out that face masks that meet international standards, such as N95, provide better respiratory protection than surgical masks^35^. Masks that are more suitable for use in the operating room should be developed in the future.

A study by Kisch et al. showed that polytetrafluoroethylene (PTFE)-coated blades are effective in reducing smoke concentrations^36^. Therefore, medical institutions could consider replacing the traditional stainless steel blades with PTFE-coated blades in order to reduce the risk of exposure to surgical smoke.

The components of surgical smoke vary across different surgical sites and different surgeries. This study was limited to the otolaryngology endoscopic surgeries. The results in other department may not be the same, but the risk of exposure to surgical smoke is still need to be concerned. In this study, we only present the surgical smoke concentrations for different time points and locations, more variables need to be investigated in further studies.

## Conclusions

The increasing concentration of particulate matter while using electrocautery knives in the OR exposed the medical personnel to a high-risk environment. This study showed the correlation between surgical smoke and otolaryngology surgeries. The highest peak of particle concentration differs in different type of surgeries due to different surgical methods and positions. In this study, particulate matter and formaldehyde from surgical smoke were evaluated and their changes in concentration at different time points and different location during the operation was presented. The results of this study showed that even equipped with a qualified ventilation system, high utilization rate might also be a factor which leads to the high concentration of formaldehyde. Medical staff should pay more attention to protect themselves from the harmful smoke, because they spend a great deal of time working in this high-risk environment. The more effective strategies for exhausting and eliminating surgical smoke should be taken into consideration in all of the medical facilities.
